# Saliva: Physiology and Diagnostic Potential in Health and Disease

**DOI:** 10.1100/tsw.2010.38

**Published:** 2010-03-16

**Authors:** Sebastien J. C. Farnaud, Ourania Kosti, Stephen J. Getting, Derek Renshaw

**Affiliations:** ^1^ Inflammation and Infection Group, School of Life Sciences, University of Westminster, London, UK; ^2^ Dr. Hadwen Trust for Humane Research, Hitchin, UK; ^3^ Division of Carcinogenesis, Biomarkers and Epidemiology Lombardi Comprehensive Cancer Center, Georgetown University, Washington, DC, USA

**Keywords:** biomarkers, saliva, oral disease, diagnostic markers

## Abstract

Saliva has been described as the mirror of the body. In a world of soaring healthcare costs and an environment where rapid diagnosis may be critical to a positive patient outcome, saliva is emerging as a viable alternative to blood sampling. In this review, we discuss the composition and various physiological roles of saliva in the oral cavity, including soft tissue protection, antimicrobial activities, and oral tissue repair. We then explore saliva as a diagnostic marker of local oral disease and focus particularly on oral cancers. The cancer theme continues when we focus on systemic disease diagnosis from salivary biomarkers. Communicable disease is the focus of the next section where we review the literature relating to the direct and indirect detection of pathogenic infections from human saliva. Finally, we discuss hormones involved in appetite regulation and whether saliva is a viable alternative to blood in order to monitor hormones that are involved in satiety.

## SALIVA AS A PHYSIOLOGICAL FLUID

The realisation of saliva as an essential, noninvasive diagnostic tool is increasing. An understanding of the physiology of the oral cavity is necessary if we are to understand the origin of the analytes contained in whole saliva. This review will discuss the composition of saliva and its main functions in terms of host defence and, more specifically, the protection saliva affords the oral cavity. We will then explore the current literature regarding saliva as a diagnostic indicator of local, oral disease and finally its role as an indicator of systemic disease.

### Regulation of Saliva Release

There are three paired extrinsic salivary glands in humans: the parotid, submandibular, and sublingual glands, as well as hundreds of smaller intrinsic salivary glands that are distributed throughout the oral cavity, including the tip of the tongue. The main intrinsic glands consist of blind-ended ducts surrounded by epithelial acinar cells. Salivary flow is under direct and indirect control from both the parasympathetic (PNS) and sympathetic nervous systems (SNS). PNS innervation is via the cranial nerves; specifically, the parotid glands are supplied by the glossopharangeal nerve (CN IX) via the otic ganglion. The facial nerve (CN VII) supplies PNS innervation to the submandibular and sublingual salivary glands via the submandibular ganglion. PNS input is dramatically increased upon food ingestion, causing enhanced serous (water-rich) saliva to be released from serous/acinar cells. SNS input to the large salivary glands is via fibres in the T1-T3 region and results in an increase in mucin-rich saliva from mucous cells. Dramatically increased sympathetic stimulation can lead to reduced salivary flow and dry mouth. Interestingly, the histological composition of the major extrinsic glands differs dramatically, with the larger parotid glands only containing serous cells and contributing around 25% of total saliva, the submandibular glands, which account for approximately 70% of total salivary output, contain around equal serous and mucous cells, and finally, the sublingual glands contain mainly mucous cells and account for only 5% of total salivary output[1,2].

## Composition of Saliva

Over time, more substances and blood-borne factors have been detected in saliva, adding to its repertoire of analytes. However, saliva is mainly composed of water (95–99.4%) and various minerals, electrolytes, hormones, enzymes, immunoglobulins, cytokines, and other components whose abundance is dependent upon the gland from which it is secreted. Whole saliva is not a homogeneous fluid, but is made up of secretions from a number of sources, predominantly the extrinsic glands, but also fluids from the intrinsic glands, epithelial cell secretions, and the gingival crevicular fluid. Blood capillaries, which pass through the salivary glands, facilitate the entry of analytes from the systemic circulation into saliva[1,2,3].

Many steroid hormones in the circulation, e.g., cortisol, are predominantly bound to large proteins, such as corticosteroid binding globulin (CBG) and albumin. This prevents the rapid entry of large quantities of steroid across cell membranes, which would otherwise lead to inappropriate stimulation of steroid receptors. This explains why only the *free*, biologically active, steroid is found in saliva. Protein-bound and sulphated steroids, such as dehydroepiandrosterone sulphate (DHEAS), are prevented from diffusing directly through the acinar cells and must pass through the tight junctions between the acinar cells of the salivary glands. This restriction leads to reduced concentrations of DHEAS in stimulated saliva samples compared to unstimulated samples, due to its dependency on flow rate[4].

## Functions of Saliva

Evidence from patients with salivary hyposecretion disorders, such as Sjögren’s syndrome, suggests that saliva is essential for the normal mastication of food. Eating can be difficult and uncomfortable due to a lack of salivary lubrication. As well as providing lubrication, saliva (or the products it contains) prevents bacterial expansion and reduces tooth decay[5].

Digestive components include α-amylase, which is involved in the conversion of polysaccharide carbohydrates, such as starch, into the polysaccharides maltose and dextrin, and lingual lipase secreted from the intrinsic salivary glands. Lingual lipase initiates the breakdown of lipids prior to food entry into the duodenum and works optimally at low pH[6].

## Immune Defence

Increasingly, factors identified as having a role in a host defence now outnumber the known digestive factors, highlighting the need for a disease-free oral cavity in order to maintain adequate nutritional intake.

Salivary defence mechanisms are numerous and include locally and systemically produced immunoglobulins, lysozyme, mucins, and an array of antimicrobial peptides (AMPs). Immunoglobulins were first discovered in saliva in the 1960s[7] and IgA, not IgG, was found to be the dominant form[8], unlike in the systemic circulation where IgG is more abundant. Subsequently, IgA was found to be the dominant immunoglobulin in all mucous secretions[9]. Unlike systemic IgA, which exists as a monomer, salivary IgA was found to be polymeric, consisting mainly of dimers[10]. These dimers of secretory IgA (sIgA) are attached by a hinge protein, named the J chain, produced *in situ* in IgA-secreting plasma cells residing in the stroma of salivary glands[11]. This complex is then transported across the epithelial membrane by a 100-kDa glycol-protein-named membrane secretory component or polymeric Ig receptor (pIgR), which allows this immune dimer to diffuse through the epithelial cells. The membrane secretory component is located on the basolateral surface of serous-type glandular epithelial cells and is partly (the 20-kDa C-terminus) degraded upon exocytosis from the apical epithelial membrane. On release into the mucus-rich lumen of the oral cavity, the remaining 80-kDa form of the membrane secretory component serves to resist proteolytic cleavage of the sIgA dimeric complex[12,13]. Once secretory IgA is released into the oral cavity, it acts as a first line of defence in the innate immune arsenal by immobilising antigens such as bacteria. Monomeric IgG from the systemic circulation also enters saliva, not through the salivary glands, but via gingival crevicular fluid[14]. IgG, which does enter the oral cavity via the salivary glands, does so without the aid of the membrane secretory component and therefore must pass through the epithelium in a paracellular fashion and in levels therefore dependent upon the state of the gingival membrane[15].

Lysozyme describes a family of strongly cationic, antimicrobial enzymes that cause damage to the cell walls of bacteria by hydrolysis and are common to mucosal secretions, such as saliva and tears. However, Gram-negative bacteria, which contain lipopolysaccharides (LPS) in their cell walls, are largely resistant to this type of agent[16]. Mucins are large glycoproteins that are highly viscous and relatively insoluble, two highly desirable qualities for a substance charged with maintaining membrane integrity under harsh conditions[17]. Mucins therefore protect the soft tissues of the oral cavity from dehydration and mechanical damage. Mucins are also responsible for the selective adhesion of bacterial and fungal agents and may help to prevent biofilm formation. They also protect both soft and hard tissues from abrasion damage by forming a lubricating coating on tissues[18].

Salivary peroxidise (or sialoperoxidase) acts as a catalyst for the oxidation of the salivary thiocyanate ion into hypothiocyanate, which has antimicrobial activity[6,19,20].

## Antimicrobial Peptides

As part of our immune response, AMPs, a family of antimicrobial agents, have emerged over the last decades as a major component of the innate defence mechanism. AMPs have been identified in most physiological fluids and in an exposed environment, such as the mouth, where they play an essential role in the balance between health and disease[21]. Although AMPs, also referred to as host defence peptides (HDP) or “alarmins”, have now been identified in most organisms, including bacteria, fungi, plants, insects, amphibians, and mammals, including humans, they have been the focus of scientific interest only over the last 30 years.

One of the first insights into innate immunity was published in 1972 by Boman et al.[22], where it was shown that *Drosophila* flies could be protected from a lethal dose of bacteria after an injection of harmless bacteria[22]. These results indicated the existence of a cell-free immune response, different from the mammalian immune system that produces antibodies. The antimicrobial agent was only isolated 8 years later from a larger insect, the Cecropia moth, from which derived the name of the first family of AMPs, the cecropins[23]. The first disulfide bridge containing peptides were identified by Lehrer’s group[24] 3 years after the isolation of the cecropins, which would later become the large family of the defensins, but decades would pass before the concept of AMPs would be recognised. Today, several databases have gathered the continuous identification of these peptides, with the Antimicrobial Peptides Database of the University of Trieste comprising over 1,000 peptides.

Generally, AMPs designate mainly cationic AMPs, therefore positively charged, although negatively charged AMPs have also been described[25]. Contrary to classical antibiotics, AMPs are ribosomally synthesised and therefore genetically encoded. Although AMPs were thought to represent a single group of molecules originally, their ever-increasing number has made the task of grouping AMPs under one definition more difficult. Their primary sequences can be very different, ranging from 10 to 50 amino acids in length, although longer molecules have now been proposed to belong to this group. Cationic AMPs contain a mixture of positively charged residues, such as arginine, lysine, or, in acidic environments, histidine, and a large proportion of hydrophobic residues[26,27]. Generally, AMPs are classified into four groups based on their secondary structure content: (1) peptides containing extended structures that form α-helices, (2) peptides characterised by a β-sheet structure due to the presence of disulphide bonds joining β-strands, (3) β-hairpin or loop formed via the presence of a single disulphide bond that can lead to cyclisation of the peptide chain, and (4) extended structures often with a predominant amino acid (Fig. 1). A common feature of all systems studied so far is that AMPs are all issued from longer prepropeptide precursors, where the so-called prosequence must be proteolytically cleaved from the N-terminus to form the active peptide structure (Fig. 2). AMPs can either be continually secreted from organisms or expressed as a response to a specific stimulus. In numerous cases, the proform of the peptide is stored as an inactive form until required[28]. In other cases, the precursor of the peptide can be a larger protein that fulfils a different function, such as the lactoferricin derived from lactoferrin[29]. The classification of these peptides is not always easy since most of these peptides are unstructured in free solution and only fold into their active configuration upon interacting with biological membranes. This polymorphism is the base of their mode of action.

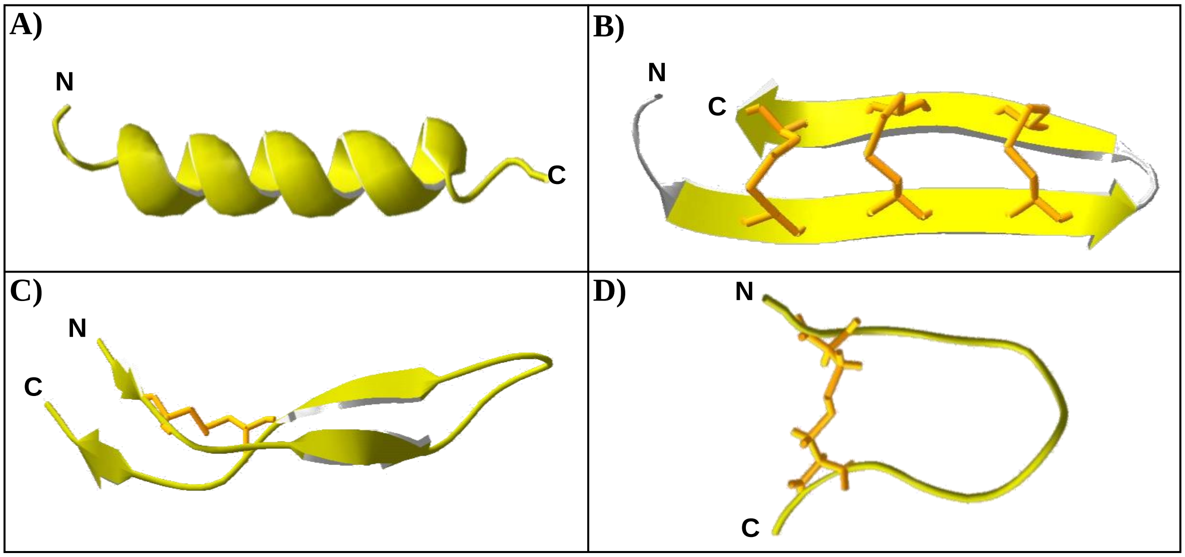

**FIGURE 1.** (A) The α-helical peptide pleurocidin from the mucus membranes of winter flounder. (B) Retrocyclin-2, a circular minidefensin with significant potential as an agent against HIV, influenza A, and herpes simplex virus, whicht forms a β-hairpin braced by three disulphide bonds that defines a cystine ladder motif. (C) Bovine lactoferricin (LfcinB), a 25-residue AMP released by pepsin cleavage of the 80-kDa iron-protein lactoferrin that reveals a distorted antiparallel β-sheet. (D) Indolicidin-derived peptide CP-11, an amphipathic molecule with a U-shaped backbone bringing the N- and C-termini, and cyclised through a disulphide-bonded peptide.

Despite their structural diversity, AMPs tend to be amphiphilic, with both hydrophobic and hydrophilic domains[30]. In a two-step mechanism, the positively charged residues are responsible for the first part of the interaction of AMPs with negatively charged membranes, as described with LPS of Gram-negative

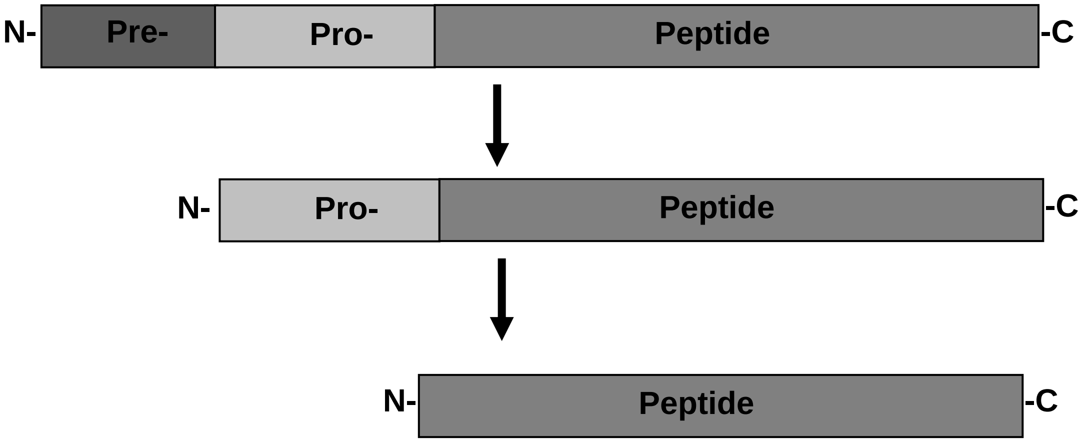

**FIGURE 2.** A pre- or signal sequence N-terminal to the prosequence provides targeting to an intracellular membrane structure and must be post-translationally cleaved to form the inactive propeptide that can be stored. Whereas in eukaryotes the target is the endoplasmic reticulum, in bacteria, it is the cytoplasmic membrane.

bacteria, which is then followed by the insertion into the hydrophobic interior of membranes[29]. Their interaction with membranes is an essential feature of AMPs, although not necessarily their final target, but the full mechanism of AMP-mediated cell death is not fully understood beyond that first step[28]. It has been proposed that following cell entrance, DNA and RNA binding may occur, leading to cell death[31]. However, most studies have focused on their mode of action at the membrane level, where AMPs are suspected to align on the cytoplasmic membrane surface and after accumulation, reorient themselves perpendicular to the membrane surface. Several modes have been proposed to describe this membrane interaction with the “barrel stave” model, the “toroidal pore” model, and the “carpet-like” model[28]. The common factor of the three models is the transitory formation of pores leading to the loss of the permeable barrier, accompanied by the loss of cellular integrity, i.e., pH, salt, and electrical gradient.

AMPs can be found secreted at the epithelial surface of the site of infection, but can also originate from the major source of AMP in the body, the neutrophils, which harbour, amongst other defensive agents, the two largest families of mammalian AMPs, cathelicidins and α-defensins.

### *Cathelicidins*

Members of the cathelicidin family of antimicrobial polypeptides have been isolated from many different species of mammals[32]. They are characterised by a highly conserved N-terminal cathelin domain and a highly variable C-terminal cathelicidin peptide domain that varies among species, yielding multiple peptides with a variety of sizes, sequences, and structures, but with only one described in humans, hCAP18. It is that precursor which, after cleavage, generates the AMP LL-37 that has been shown to be active against bacteria, viruses, and fungi[33,34]. It has been found expressed not only by neutrophils and epithelial tissues lining the oral cavity, but also in the respiratory, urogenital, and gastrointestinal tracts[35,36,37]. In addition to its antimicrobial function, LL-37 has been shown to be a multifunctional effector molecule capable of not only killing pathogens, but also modulating the immune response and promoting wound healing with chemotactic properties for monocytes, T cells, neutrophils, and mast cells, as well as stimulating mast cell histamine release[37,38,39,40].

The absence of LL-37 in the saliva of patients with Morbus Kostmann indicates that saliva could be a convenient mirror of granulocyte components[41]. Morbus Kostmann is a severe, recessive disorder described by Kostmann in 1956[42]. Kostmann patients are born with massively reduced granulocyte numbers and traditionally afflicted children die from bacterial infections during their first year of life.

Since 1990, daily injections of cytokines led to the survival of patients with restored granulocyte levels; however, oral health problems persist[43]. Western blot analysis revealed that LL-37 was absent both in granulocytes and saliva. One patient was cured with a bone-marrow transplant, which restored LL-37 levels and saw the return of good oral health for this patient; therefore indicating the fundamental role played by LL-37[41].

## Defensins

Defensins are amongst the first AMPs to be identified[44]. They are small (15–20 residue), cysteine-rich cationic AMPs containing three pairs of intramolecular disulphide bonds, found in both vertebrates and invertebrates, with a wide spectrum of activity. Three forms of mammalian defensins have been identified and classified on the basis of their pattern of disulphide bonding: α-defensins, β-defensins, and the cyclic θ-defensins, the last one being quite rare and having only been found in rhesus macaque leukocytes. Although the α- and β-families do not share sequence similarity, they are encoded by adjacent genes and have been proposed to have evolved from a common premammalian defensin gene[45]. Since both α- and β-defensins have been found to have reduced antimicrobial activities in the presence of a physiological concentration of salt, their direct antimicrobial effect *in vivo* was proposed to be mainly where there is low ionic strength; therefore, in phagocytes and on the surface of skin and mucosal epithelia.

α-Defensins have been identified in humans, monkeys, and several rodent species, and are expressed primarily in neutrophils as well as macrophages and the Paneth cells of the intestines. Due to their relatively low direct antimicrobial activity against most of the oral microbes tested, recent research has focused on their indirect antimicrobial mode of action. It was suggested that α-defensins cannot function as antibacterial molecules by themselves, but can synergistically work with cathelicidins to exert the antibacterial activity in the extracellular milieu by augmenting the membrane permeabilisation of target cells[14]. Human neutrophil-derived α-defensins (HNPs) have been shown to attract CD4+ T cells and immature dendritic cells by using chemokine receptors[46], to stimulate mast cell degranulation[47] and to regulate complement activation[48]. Furthermore, a role of inhibitor of the production of immunosuppressive glucocorticoids by competing against the adrenocorticotropic hormone for its receptor binding has also been suggested for α-defensins

Whereas α-defensins are released by exocytosis from neutrophils, β-defensins are secreted by epithelial cells at the site of microbial colonisation in response to inflammatory products such as LPS or proinflammatory cytokines. The first lingual antimicrobial peptide (LAP) was a β-defensin identified in a bovine oral epithelium that showed a broad spectrum of antibacterial and antifungal activities. It was later also found in salivary glands and saliva. The increased level of expression of LAP in the epithelium surrounding naturally occurring tongue lesions coincided with the acute and chronic inflammation in the underlying lamina propria, and supported a role for epithelial AMPs as integral components of the inflammatory response[49]. Human β-defensins (hBDs) are expressed in all human epithelial tissues[44] and in the mouth have been identified in gingiva, tongue, salivary glands, and mucosa[50]. Variations of the levels of human defensins were detected in the saliva of patients with oral inflammation and oral carcinomas[51,52], where hBD-1 seemed to be constitutively expressed, and hBD-2 and hBD-3 were up-regulated in inflamed skin and other epithelia. In carcinoma, the decreased level of hBD might increase bacterial susceptibility[53].

In contrast to normal epidermis, trachea, and gut, not only the constitutive hBD-1, but also the inducible hBD-2 were found in all gingival biopsies tested, both healthy uninflamed and inflamed samples[54,55]. It was proposed that such an exposure was due to the presence of commensal bacteria, providing an advantage for the presence of potential pathogenic organisms. The presence of the three variants was justified by a different role for each hBD, where hBD-1 may prevent commensal bacteria from becoming opportunistic pathogens, and hBD-2 and hBD-3 may be more effective against pathogens.

Evidence suggests a strong relationship between periodontal inflammatory disease and systemic diseases such as cardiovascular disease, and periodontal disease is now recognised as a major public health problem in need of a tight control of its microbiological fauna[56]. The junctional epithelium at the attachment of the soft tissue to the tooth surface is a very vulnerable surface and therefore a site of secretion of β-defensins, but the presence of neutrophils that migrate through the junctional epithelium provide additional protection through the release of α-defensins and LL-37[57]. To provide a complete barrier to microbial colonisation, both neutrophil and epithelial AMP complement each other in the oral cavity. In such a system, the analogy with the intestine cannot be ignored, where the crypts are protected by α-defensins and the epithelia of intestinal villae express β-defensins[58].

## Adrenomedullin

Another peptide known to be expressed by many surface epithelial cells that was also identified in oral epithelial cells is the vasoactive peptide adrenomedullin, a multifunctional peptide that has been recognised to have antibacterial function against both Gram-positive and Gram-negative bacteria[59,60]. Adrenomedullin is a 52-amino-acid peptide with one intramolecular disulphide bridge, which although homologous to the β-defensins, has a different structure. Although its antimicrobial properties are now well defined, its analogy with calcitonin gene-related peptide, together with its interaction with the calcitonin receptor-like receptor, led some to suggest that it could have hormone-like functions in the control of the circulation[61].

## Histatins

The histatin family comprises a group of 12 histatins and eight derived peptides, all containing a high number of histidine residues, and are secreted by the parotid and submandibular glands[62]. Histatin 1, 3, and 5 are the major histatins detected in human saliva. Their primary sequence shows a high degree of identity, with a positive net charge, and they have been proposed to be implicated in several biological processes in the maintenance of the oral cavity[63]. Specifically, histatin 1 was shown to inhibit hydroxylapatite crystal formation and was proposed to maintain the surface integrity of enamel[64]. Later, the direct antimicrobial effects of histatins were investigated and shown to be limited to *Candida albicans,* with histatin 5 being the most potent[65]. Together, these data suggested that histatins in parotid and submandibular gland secretions play a major role in the primary innate defence mechanism, and the presence of a phosphorylated serine in histatin 1 suggests that it could be a precursor of the acquired enamel pellicle. The concentration range found in saliva corresponds to its minimal inhibitory concentration (MIC), but was found to decrease between the ages of 45 and 75.

## Lactoferrin

Lactoferrin (Lf), the red moonlighting protein, is a glycoprotein that belongs to the transferrin family that was first identified as the red iron-binding protein in bovine milk by Sorensen and Sorensen in 1939[66]. The protein was later found, not only in milk, but also in various exocrine secretions, including saliva, and various tissues[30]. Although the protein was first thought to play a role in iron metabolism, its role as an iron carrier is now questioned and numerous other functions have been proposed[67]. Amongst the multiple functions proposed for Lf, its antimicrobial activity is one of the least controversial and although several modes of action have been suggested, an iron-scavenging function that prevents microbial growth is the most accepted.

Lf was found to be present in saliva, with a marked elevation in the parotid concentration during the active phase of chronic recurrent parotitis[68], and inflammatory stimulation of Lf expression suggested a basic protective mechanism in exocrine glands towards iron scavenging and microbial growth prevention. Its ability to sequester iron is also found in other transferrins, but its different biophysical properties seem to confer on Lf a stronger affinity and the ability to retain iron even in acidic conditions. Deprivation of iron has also been shown to prevent the formation of biofilms for *Pseudomonas aeruginosa*, although since the concentration of Lf used was far less than that required to inhibit the growth by bacteriostatic mechanisms, the sequestration of iron by Lf might interfere with a more complex mechanism than the growth need of the bacteria[69]. A similar effect of iron scavenging could be present in the oral cavity.

Paradoxically, Lf has been found to have not only antibacterial, but also probacterial properties. Although one of the most common mechanisms in iron acquisition by bacteria involves the synthesis and secretion of small iron-chelating molecules called siderophores, some highly adapted Gram-negative species have developed a simplified and more specific variant of these uptake systems that by-passes the siderophore. In these pathways, the iron is directly removed from the transferrin or Lf by distinct specific receptor complexes located on the outer membrane[70]. In *Neisseria meningitidis*, receptors for both Lf and transferrin, lactoferrin-binding protein (Lbp) and transferrin-binding protein (Tbp), respectively, have been identified. Another such example of evolutive bacterial prowess is in *Helicobacter pylori*–associated iron-deficiency anaemia (HPIDA), where Lf sequestration of iron contributes to iron deficiency and where the antimicrobial function of sequestering iron is turned into the promicrobial function of providing iron, illustrating the aptitude of micro-organisms to adapt and evolve even in the most difficult situation[71]. Such a balance is well illustrated in the oral cavity with *Actinobacillus actinomycetemcomitans* (Aa), a facultative Gram-negative rod that is associated with localised aggressive periodontal disease in juveniles (LAgP), endocarditis, and other focal infections. Lf has been shown to kill Aa in its iron-free form (apo) and reduce binding to host cells in its iron-saturated form (halo)[72]. However, opposite results were obtained *in vitro*, showing that neither did Lf kill clinical isolates of Aa, nor did Lf, with reduced levels of bound iron, interfere with its attachment. Together, these results suggested that Lf with low iron levels could promote colonisation of Aa[73]. This suggests that, as in the gut, Lf in saliva might have antithetical functions.

In the intestine, the presence of lactoferricin, a 47-residue AMP resulting from the N-terminal cleavage of Lf, has been suggested. The existence of this peptide was demonstrated in 1992 by Bellamy and colleagues following pepsin hydrolysis of bovine and human L[74]. Although no iron-binding capacities could be identified, both fragments showed enhanced antimicrobial activity, suggesting another mode of antimicrobial activity. So far, the presence of lactoferricin in the oral cavity has never been shown, but the analogy between the oral cavity and the intestine found for all other antimicrobials suggests that this is a possibility.

### *Calprotectin*

Calprotectin, also called MRP-8 and MRP-14, CFA or calgranulin A, is a calcium and zinc protein composed of two subunits of 8 and 14 kDa, and is released by neutrophils in the biological fluids under inflammatory states such as periodontal diseases. It was found in blood and interstitial tissue fluid in several infectious, inflammatory, and malignant disorders, and expressed in cells of stratified oral epithelia and in cultured gingival epithelial cells[75]. Its role as an antimicrobial was therefore proposed, suggesting that it might act by depriving micro-organisms of zinc[75]. As for the AMPs, an analogy with the intestine can be emphasised since calprotectin can be detected in faeces, where it represents a diagnostic tool for inflammatory bowel disease.

## Oral Tissue Repair

Oral tissue repair is known to be accelerated compared to dermal wound healing, suggesting the presence of substances in saliva that accelerate re-epithelialisation of oral epithelial cells. Epidermal growth factor (EGF) has a role in cell growth and proliferation, and is present in human saliva[76], as is the EGF receptor, which was identified on human buccal mucosa[77]. These observations indicate a possible role for EGF in human oral tissue repair. In rodents, salivary EGF is produced in large quantities; however, in human saliva, the levels of EGF are approximately 100,000 times lower[78] than in the mouse, suggesting that other factors may be of importance in human oral tissue repair. Histatins are a family of histidine-rich proteins (called HRPs) originally isolated in parotid saliva and have antimicrobial activity (see above section)[64,79]. Recent publications suggest that histatin-1 and histatin-2 extracted from human saliva enhanced epithelial migration in an *in vitro* epithelial cell study[80,81], suggesting a dual role for these proteins as antimicrobial agents and substances involved in enhancing tissue repair.

### Saliva vs. Serum Sampling

Saliva offers increased flexibility, cost effectiveness, convenience, and is less invasive compared with serum sampling. The collection of saliva is noninvasive and negates the need for trained medical staff. This allows the collection of biological material in a stress-free, painless, and economically viable manner. Increasingly, for studies involving the collection of steroid hormones *only*, participants/patients can produce multiple samples whilst in the comfort of their home environment. Samples can then be stored at ambient temperatures, allowing transportation by regular post and, therefore, reducing costs and inconvenience. Increasingly, saliva is being chosen as the biological medium of choice in studies involving children[82]. Saliva also presents less risk of infection to the technical staff involved in the processing and assaying of biological samples compared with blood due to the reduced presence of antigens, e.g., HIV virus[83]. Patient compliance will remain a difficult hurdle to overcome for studies that require timed samples; for example, to assess the awakening cortisol response (ACR), which is now used extensively in psychosocial studies measuring salivary cortisol[84,85].

## SALIVA AS A BIOMARKER OF ORAL DISEASE

Much of the early research on saliva focused on its local biomarker function, as an indicator of periodontal health. Here we will not discuss periodontal health as this has been extensively reviewed elsewhere[86].

### Oral Cancer

Oral cancer, predominantly oral squamous cell carcinoma (OSCC), is the most frequent malignant tumour in the head and neck region affecting over 300,000 people worldwide per year[87]. According to the American Cancer Society, in 2006, oral cancer represented ~3% of all malignancies in men and 2% of all malignancies in women. Although oral cancers have broadly varying rates of incidence and mortality around the world, smoking/chewing tobacco and alcohol consumption account for the vast majority of the disease burden worldwide[88,89]. However, a small proportion (15–20%) of oral cancers occurs in nonsmokers and nondrinkers, suggesting the presence of other risk factors. Over the past 15 years, epidemiologic and molecular data suggest that human papillomavirus (HPV), the necessary cause of cervical cancer, may promote carcinogenesis in the head and neck region[90,91].

OSCC tumourigenesis progresses through a series of histopathological stages from hyperplasia to dysplasia of varying degrees, and to carcinoma *in situ* prior to development of invasive squamous cell carcinoma[92]. Patients with oral cancer often present with symptoms at a late stage and delayed detection is likely to be the primary reason for the poor prognosis associated with the disease. Despite refinement of surgical techniques and adjuvant therapies, it has been estimated that 26–47% of patients will develop a recurrence within 2 years of surgical resection and have an annual 5% chance of developing a second primary tumour[93]. Moreover, because of the location of the tumours in the head and neck region, patients often encounter post-treatment defects and functional impairments[94].

Identification of a biomarker that complements clinicopathological findings could help to screen patients at risk, predict disease outcome, and effectively help to plan treatment strategies. For obvious reasons related to saliva being the proximal fluid for oral cancer, together with availability and minimal invasiveness associated with saliva collection, salivary biomarkers are good candidates for oral cancer detection and monitoring of disease progression.

The majority of oral cancer biomarker development has focused on the tumour-suppressor protein TP53. Mutations in the p53 gene are considered to be the most frequent genetic alterations found in human cancer, including OSCCs[95], especially those of advanced stages[96], and it is a result of both genetic and environmental carcinogenic influences, such as tobacco smoke and alcohol[97]. Tumour-specific p53 mutations were identified in 71% of saliva samples from patients with head and neck cancer, and it was estimated that for every 100 cells isolated from saliva, two to three cells had exfoliated from the patients' cancer and contained a p53 mutation[98]. Since mutant p53 has a prolonged half-life, it leads to a very high accumulation of inactive TP53 protein in the nucleus of the tumour cell[99,100,101] and this leads to the immune system producing antibodies against TP53, which can be detected in the sera of cancer patients[102,103,104], but not healthy individuals. It is estimated that approximately one-third of patients with a p53 mutation have TP53 antibodies in their serum[105,106]. In oral cancer, accumulation of TP53 antibodies is believed to be an early event in nondetectable neoplastic lesions[107], and quantification of TP53 antibodies may be used as a prognostic indicator of disease severity and patient outcome[108,109]. The presence of TP53 antibodies in saliva has also been reported, although the correlation with seropositivity was around 50%[109]. Whether this reflects a true correlation or differences in performance of the ELISA procedure remains to be elucidated.

A small study of OSCC patients and a healthy comparison group demonstrated that the amount of DNA recovered from saliva was quality and quantity suitable for polymerase chain reaction (PCR) amplification and suggested that an exonic mutation of the p53 gene in saliva might be a molecular marker for the disease[110]. Many genes are induced by the expression of the TP53 protein[111]; therefore, their contribution to cancer development warrants attention. Immunocytochemical evaluation of TP53 and the antiapoptotic protein bcl-2 showed an association between expression of these two proteins and increasing histological abnormality in a high-risk population. More specifically, although expression of TP53 and bcl-2 was absent in hyperplastic leukoplakia lesions, both proteins were expressed in leukoplakia with apparent dysplasia and the levels of expression were higher in almost all invasive cancer lesions studied[112]. Targeting the bcl-2 gene with gene therapy could be a promising way to prevent growth of oral cancer cells, as shown in an *in vitro* study[113]. The TP53 protein also seems to have linked transduction pathways with EGF and its receptor[114]. EGF and its receptor exert mitogenic activities in epithelial cells[115]. Preliminary results showed a lower EGF concentration in saliva of presurgery patients and its growing tendency postsurgery, suggesting a role of this factor in oral cavity carcinoma development[116]. EGF receptor is generally overexpressed in oral cancers and correlates with aggressive tumour behaviour[117,118,119,120]. Although its strength as a potential salivary biomarker and therapeutic target for oral cancer needs to be investigated with great care, the confounding effect of smoking needs to be addressed[121].

High-risk HPVs (e.g., HPV 16 and HPV 18) are known to be tumourigenic in human epithelial tissues; however, simple detection of HPV DNA in tumour biopsies is not sufficient for evidence of causation in cancers of the oral cavity. Integration of HPV DNA into the human cellular genome is an important step for malignant transformation. Molecular studies have shown that high-risk HPV integration results in production of the viral oncoproteins E6 and E7, which promote tumour progression by inactivating the p53 gene and retinoblastoma tumour-suppressor gene products, respectively[122,123,124,125]. Using a qualitative PCR method, detection of HPV DNA in saliva rinses was examined and its association with HPV in tissue specimens was reported. Although one study showed that there was no association between salivary and tissue HPV presence[90], a study by Smith and colleagues[126] found that HPV high-risk types detected in oral exfoliated cells were predictive of HPV high-risk types in tumour tissue. Real-time PCR-based methods have proven to be more sensitive compared to other conventional methods in determining the HPV DNA level in human specimens[127,128,129] and were also recently evaluated in saliva rinses[130]. It was reported that some tumours that were HPV 16 positive did not yield HPV DNA positivity in saliva rinses and that the saliva rinse level of the HPV DNA was invariably lower than that of the tumour, presumably due to a diluting effect[130]. Moreover, the method seemed to be working more effectively when the HPV E7 DNA was detected compared to HPV E6 DNA, probably due to improved sensitivity associated with the E7 probe and primers.

Endothelin 1 (ET-1) is a vasoactive peptide normally synthesised by human keratinocytes. Together with its role in the development and progression of vascular disorders such as hypertension, it has been found to be overproduced by a number of malignancies[131,132,133]. The fact that ET-1 appears to be relatively stable in saliva allowed the establishment of an ELISA-based method for measuring salivary ET-1[134,135] that was used by recent pilot study in oral cancer. The group demonstrated a 3.5-fold increase of the peptide among oral cancer patients compared to healthy subjects[136]. Larger studies are needed to confirm the observation, as well as studies designed to test the correlation with tissue levels and disease severity. Similar to the potential role of ET-1 as a salivary peptide that can be used to diagnose oral cancer, the role of defensins in the oral cavity environment has been explored. Defensins are cysteine-rich cationic proteins contained in cytoplasmic granules of polymorphonuclear (PMN) leukocytes with both antimicrobial and cytotoxic functions in the oral cavity[137]. Using reversed-phase HPLC, human α-defensin-1 (HNP-1) was identified as the peptide to be significantly elevated in the saliva of six patients with OSCC compared to saliva from patients with adenocarcinoma and among healthy volunteers; however, it dropped in OSCC patients by about 50% following surgery[138]. Circulatory epithelial tumour markers, such as Cyfra 21-1, tissue polypeptide antigen, and CA125, have also been investigated in the saliva of OSCC (tongue) patients and have been found to increase significantly (approximately fourfold) with disease[139].

It is now recognised that the mixture of cytokines that is produced by the tumour microenvironment can act to alter tumour development and progression[140]. The role of inflammatory cytokines, specifically interleukin-6 and -8 (IL-6 and IL-8), has been examined by several groups in relation to oral cancer biomarker development. Both cytokines were found to be significantly elevated in the saliva of patients with oral cancer compared to healthy controls, with IL-6 being undetectable in the saliva of control subjects[141]. IL-8, but not IL-6, was found to be significantly higher in the saliva of early-stage OSCC patients compared to controls of the same age and sex selected because of comparable smoking histories to the cases[142]. The role of IL-8 as a biomarker for oral cancer was also suggested by a microarray study performed on circulating RNA in the saliva of 32 patients with OSCC compared with healthy controls[143]. However, as discussed in a recent review[144], one has to be cautious when interpreting results associated with inflammation-related genes in a case-control design study, and addition of an inflammatory group (e.g., periodontal disease) that is cancer free can reveal whether the alterations in the cytokine profile are cancer-related alterations.

Another cytokine expressed and secreted by salivary glands into saliva is leptin, a hormone originally identified as a regulator of food intake and energy expenditure[145]. Since it was first introduced, leptin has gained increasing attention as a factor involved in cancer development[146,147]. A recent study investigated the expression of leptin and its receptors in parotid salivary gland tissue isolated from patients with cancer and noncancer patients that underwent surgery for other indications. Results showed that in all salivary gland tumours, leptin was expressed in much higher amounts than in healthy parotid tissues and that the receptors were up-regulated with cancer state. Salivary leptin measurements agreed with the observation in tissue showing a five- to sixfold increase in concentration among oral cancer patients[148].

Taking a different approach to studies that examine levels of secreted factors in different biospecimens, researchers have been interested in epigenetic changes of tumour DNA. An epigenetic pathway of transcriptional inactivation for many tumour-suppressor genes includes CpG island hypermethylation within promoter regions[149,150,151] that can be detected by using quantitative methylation-specific PCR (Q-MSP)[152,153]. The method has proven effective in detecting aberrant promoter methylation in the sputum of patients with squamous cell lung carcinoma up to 3 years before clinical diagnosis[154], and has been tested in saliva samples of head and neck cancer patients using a panel of 2-20 genes[155,156,157,158,159] selected from their known role in head and neck carcinogenesis. More recently, a genome-wide methylation array analysis was reported on both pre- and postoperative saliva in order to discover new genes that could be part of a reliable gene classifier[160]. Microsatellite analysis can reveal either loss of heterozygosity (LOH) or microsatellite instability (MI) in the amplified microsatellite repeat locus, and these alterations have been used as markers of clonality and to detect cancer cell DNA in a background of normal cells[161]. Microsatellite analysis of tumour-specific genetic alterations in saliva has been investigated as a potential marker of oral cancer detection. Microsatellite instability was detectable in 24 out of 25 cases with cancer, and LOH was identified in 19 out of 31 cases, while no microsatellite alterations were detected among the healthy comparison group[162].

The role of salivary proteomics for oral cancer biomarker discovery has been examined in a pilot study. Several salivary proteins were found to be present at differential levels between patients with oral cancer and five candidates were successfully validated using immunoassays on an independent set of cancer patients and subjects without oral cancer[163]. A mass spectrometry method was developed for whole saliva glycoprotein identification[164] and it was later demonstrated that oral fluid contains proteomic signatures that may serve as biomarkers for human diseases such as oral cancer[165]. Finally, microRNA signatures have attracted a lot of attention as prognostic indicators of cancer because of their role in regulating up to two-thirds of all known human genes[166]. MicroRNAs are small noncoding RNA sequences that bind to 3'UTR of target genes inhibiting initiation of translation and promoting deadenylation of target mRNAs[167,168]. Using tissue samples extracted from benign salivary gland tumours, namely pleomorphic adenomas, investigators were able to demonstrate an up-regulation of genes associated with signalling pathways involved in tumourigenesis[169]. As microRNAs are present in various clinical samples including saliva[170], they may prove to be valuable oral cancer biomarkers.

## SALIVA AS A BIOMARKER OF SYSTEMIC DISEASE

The literature concerning the area of psychosocial stress and particularly the measurement of salivary free cortisol and α-amylase are vast and specialised, and therefore beyond the scope of this review. We will concentrate on nonstress-related systemic pathologies.

It has been suggested by some that saliva can be viewed as “the mirror of the body”[171], reflecting the body’s general state of health. In fact, it is now known that many substances found in peripheral blood are also found in saliva, although generally lower concentrations are found in saliva than in blood[172]. With the emergence of new and highly sensitive technologies, it is now possible to analyse minute quantities of substances in human saliva. Proteomics technologies have allowed the determination of over 1,000 proteins in human whole saliva[173]. Interestingly, around 22% of these proteins were unique to either the parotid or the submandibular/sublingual salivary glands (http://hspp.dent.ucla.edu/cgi-bin/hspmscgi-bin/welcome_c.cgi), suggesting that there are likely to be distinct differences in functions of the exudate secreted from the major salivary glands.

The salivary transcriptome has been even more diverse than the proteome, with over 3,000 mRNAs so far identified in normal human saliva[174]. One study used four of these salivary mRNAs (*IL-8, OAZ1, SAT,* and *IL1β*) as biomarkers for the determination of oral cancer, with an overall hit rate of 91%. Crucially, for the success of saliva as a viable biological fluid for the detection of oral cancers, salivary gene expression was found to be a more sensitive indicator of predicting disease (in this case oral cancer) than blood[175]. It is hoped that distinct mRNA groups or “signatures” will be identified in the future that act as biomarkers of the major systemic diseases[176]. This approach, combined with a rapid sampling and detection system, such as the oral fluid nanosensor test (OFNASET)[177], in the future may offer rapid determination and diagnosis of major human diseases without the need for blood to be drawn from the patient, and alleviate a lengthy and anxious waiting period.

### Detection of Systemic Cancers in Saliva

Elevated levels of the cancer antigen 15-3 (CA15-3) were detected in the saliva of women diagnosed with breast cancer compared to controls[178,179]. Furthermore, in the same study, the recognised tumour marker c-erbB-2 (erb) was found to be present in the saliva of breast cancer patients and absent in control subjects, possibly representing a more robust biomarker of breast cancer than CA15-3[179]. Another cancer antigen, CA125, which is frequently used as a serum marker of ovarian cancer, was also found to be elevated in the saliva of patients with ovarian cancer. In these studies, salivary levels were found to be a better diagnostic marker of the disease than serum values[180]. A later study was able to use the diurnal rhythm in salivary CA125 levels to direct successfully a therapeutic antitumourigenic agent against ovarian cancer[181].

EGF (as discussed earlier) is present in the saliva of humans and fulfils a growth and repair role in the oral cavity. EGF overexpression is thought to be linked to tumourigenesis. Salivary EGF was found to be significantly raised in women with active and nonactive breast cancer compared to healthy women[182]. The observation that systemic diseases can influence the circadian rhythm of secreted salivary components was used in one study, where diurnal salivary cortisol measurements were used to predict breast cancer survivability using the slope of diurnal cortisol as a predictive marker[183].

## DETECTION OF SYSTEMIC INFECTIONS IN SALIVA

Infectious diseases caused by primary pathogens can be associated with high mortality rates. Therefore, the need for early detection and correct identification is essential in order to enable an appropriate treatment regime to be initiated. The exact method of detection, whether it be direct detection of the pathogen or pathogen nucleic acids (DNA or RNA), or raised host salivary antibodies to the pathogen, will vary depending on the specific pathogen. Direct detection of *Mycobacterium tuberculosis* in saliva (rather than sputum) was far less effective (98 vs. 17%) than the detection of bacterial DNA by PCR[184].

Detection of *H. pylori*, a major risk factor for peptic ulcer formation, is typically achieved by gastric mucosal biopsy, an expensive and highly invasive procedure. Reliable detection of *H. pylori* rDNA sequences by PCR in the saliva of affected patients suggests that saliva could be used for the inexpensive and routine detection of *H. pylori* bacteria in the future[185,186].

Detection of specific IgG antibodies to HIV in saliva is thought to be as reliable as in serum[187]. IgA levels may also be useful as a prognostic marker, as sIgA was found to decline as infected patients began to exhibit symptoms of the disease[188]. The presence of specific HIV IgG antibodies alone can determine whether an individual is infected and potentially contagious, although viral RNA detection and p24 antigen and combinations thereof have also be used to confirm infection[189]. Saliva sampling and testing for the determination of infectious agents, such as HIV, offers many advantages over blood, specifically the likelihood of transmission is reduced, as viral load values for HIV are thought to be lower than that of blood[190,191] and infectious virus is generally not detected in saliva[192].

Many other viruses are increasingly being detected directly by targeting DNA using PCR methodology. These include HPV[193], human herpes virus[194], herpes simplex virus[195], Epstein-Barr virus[196], measles[197], and mumps[198], although all of these viruses can also be detected by raised IgG antibodies to each virus. Some viruses, however, can only be detected directly, such as the SARS (severe acute respiratory syndrome) coronavirus[199], rabies virus[200], and Ebola[201], the latter of which has a very high mortality rate consisting mainly of antibody-negative patients[202]. Therefore, the lethality and time course of the infection may have a direct bearing on the method of detection of the disease.

## HORMONES AND APPETITE REGULATION

The modern obesity epidemic has seen a growing interest in systemic obesity markers, such as leptin and adiponectin. Leptin is secreted in humans, principally from differentiated adipocytes, and acts as a longterm regulator of body weight[203]. Adiponectin is negatively correlated with obesity, with levels *increasing* with increasing weight loss[204]. Adiponectin levels in saliva and plasma were found to be positively correlated in a group of older adults (>43 years), leading the authors of one study to suggest that salivary adiponectin levels could be used as a marker of increased risk of noninsulin-dependent diabetes and cardiovascular disesase[205]. Interestingly, it was later discovered that the collection method is critical in attaining a positive correlation between salivary and plasma adiponectin levels[206]. Leptin was first identified in saliva in 2001 and appears to be expressed at levels that correlated positively with plasma leptin[207]. It was further found to have a diurnal rhythm in saliva[208] and has since been suggested as a biomarker of oral cancers[209](as discussed earlier). The finding that leptin is produced locally in the salivary glands (confirmed by the presence of leptin mRNA and protein expression in salivary gland tissue) rather than merely being a product of the systemic circulation suggests a local role for this cytokine in oral physiology[207,208]. This theory is given further weight by the discovery of leptin receptor isoforms Ob-Ra and Ob-Rb in human parotid salivary glands, which were also up-regulated in patients with parotid gland tumours compared to control subjects[209].

Ghrelin is an orexigenic peptide hormone produced in specialised cells of the stomach and is inversely regulated by food intake. Full-length octanoylated human ghrelin was found to be significantly higher in saliva compared to plasma samples of 30 mixed-gender adults[210]. The presence of immunoreactive ghrelin protein in the ductus epithelium cells of the salivary gland was demonstrated, indicating local production of the protein was likely. The higher levels of ghrelin in saliva compared to plasma also indicated local production of ghrelin within the oral cavity[210].

Given that many of these hormones are produced locally in the oral cavity, it is tempting to speculate that they may fulfil a different physiological role than their main systemic role. Further to this, if the hormones are not reflecting systemic levels, their usefulness as a biomarker of their systemic function maybe questionable, unless the values in saliva significantly correlate with systemic levels[208].

## FACTORS AFFECTING SALIVA PRODUCTION

Normal whole saliva secretory rates vary between 800 to 1,500 ml/24-h period[211]. However, there is a wide range of factors that can lead to a reduction or an increase in the amount of saliva produced, which can then lead to serious pathology. Hypersalivation is caused by a number of conditions including various neurological disorders, including Parkinson’s disease, gastro-oesophygeal reflux disease, and hyperhydration. Heavy metal poisoning can also lead to this condition; however, the most common cause is likely to be due to the side effects of medications[211]. In particular, drugs that activate the PNS, such as physostigmine, pilocarpine, cevimeline, bethanechol, as well as antiepileptic nitrazepam, antipsychotic clozapine and lithium, and drugs used in the treatment of schizophrenia, such as risperidone, all increase saliva release.

Similarly, drugs are also primarily responsible for causing conditions of hyposalivation or xerostomia dry mouth[212]. Xerostomia and salivary gland hypofunction has a higher incidence in older people (>65years)[213], although this is not thought to be directly related to the age of the patient, but due to an increase in other pathologies and increased medication use[212,213,214,215]. Medications that lead to hyposalivation consist mainly of drugs that interfere with the actions of acetylcholine, either directly or indirectly, and include the following: anticholinergic drugs, antihistamines, antihypotensives, tricyclic antidepressants, tranquilisers, antisiezure drugs, chemotherapy drugs, and sedatives, such as β-blockers that block neurotransmission to the salivary glands[211]. A number of systemic and local pathologies can also lead to hyposalivation, as can head and neck irradiation therapy used to treat pre-existing cancers[216]. Sjögren’s syndrome is a systemic autoimmune disease that affects epithelial tissues. Primary Sjögren’s syndrome affects the lacrimal glands of the eyes and the salivary glands, leading to xerostomia and hyposalivation[217].

## CONCLUDING REMARKS

The repertoire of salivary biomarkers is ever expanding. Recently, Salimetrics® introduced a kit for measuring human salivary C-type reactive protein (CRP), which is commonly used as a biomarker of cardiovascular-related inflammation. From our own studies, we have reported that the glucocorticoid regulated anti-inflammatory substance Annexin-A1 is present in human saliva and furthermore demonstrates a positive correlation with the diurnal rhythm of salivary cortisol[218].

The success of saliva as a diagnostic media is guaranteed, particularly for substances that reflect or can be directly correlated with systemic analytes. The degree of that success is likely to depend on the introduction of mobile diagnostic devices that have the capacity to measure multiple analytes in a rapid, cost-effective manner. With many such technologies currently under development, the future of salivary diagnostics is looking very bright.

## ACKNOWLEDGEMENTS

The authors would like to thank Claire Redhead of TheScientificWorldJOURNAL for her patience throughout the preparation of this manuscript.
